# Supplementary material for: Complexome profiling of the Chlamydomonas psb28 mutant reveals TEF5 as an early PSII assembly factor
Source: Plant Cell. 2025 Mar 17;37(6):koaf055. doi: 10.1093/plcell/koaf055 (PMC12164590; doi:10.1093/plcell/koaf055)
Supplement: koaf055_Supplementary_Data [file koaf055_supplementary_data.zip › psb28_interactive.zip!/plots/intensity/qualityPlots/peptides/MDN3_Cre02.g145800.t1.2.html]

### Cre02.g145800.t1.2

Trivial name: MDN3  
SeqCoverage [median]: 32.50  
Mass: 38.11 kDa  
  
 

| Sequence | MissedCleavage | Mass [Da] | wtIntensity | muIntensity | Score |
| --- | --- | --- | --- | --- | --- |
| ALGQVAER | 0 | 842.46 | 10807.78 | 20282.02 | 157.33 |
| GTPGGSWTSMGVVSDGSYGVQR | 0 | 2183.99 | 0.00 | 1419.40 | 44.10 |
| LRVTEAELVEER | 1 | 1442.77 | 1192.37 | 3421.48 | 85.85 |
| NALEGLR | 0 | 771.42 | 2769.34 | 6970.50 | 134.66 |
| NVSIYQQQASALEANASK | 0 | 1920.95 | 0.00 | 1392.45 | 67.73 |
| NVSIYQQQASALEANASKDVK | 1 | 2263.14 | 1635.35 | 3411.47 | 61.18 |
| TGSHVGIVK | 0 | 896.51 | 0.00 | 1001.09 | 54.16 |
| VTEAELVEER | 0 | 1173.59 | 938.89 | 1633.85 | 138.98 |
| VVQGLPIDGPSR | 0 | 1236.68 | 9999.93 | 24329.78 | 126.07 |
| VVQGLPIDGPSRER | 1 | 1521.83 | 506.24 | 402.18 | 61.38 |
| VVVVANPANTNALILAENAPSIPR | 0 | 2442.36 | 1642.46 | 0.00 | 112.36 |
|  |  |  | 29492.4 | 64264.2 |  |
